# Cryopreservation and Enumeration of Human Endothelial Progenitor and Endothelial Cells for Clinical Trials

**DOI:** 10.4172/2155-9864.1000158

**Published:** 2013-09-20

**Authors:** T Bogoslovsky, D Wang, D Maric, L Scattergood-Keepper, M Spatz, S Auh, J Hallenbeck

**Affiliations:** 1 Center for Neuroscience & Regenerative Medicine, Uniformed Services University of Health Sciences, Bethesda, USA; 2 National Institute of Nursing Research, Bethesda, USA; 3 National Institute of Neurological Disorders and Stroke, Flow Cytometry Core Facility, Bethesda, USA; 4 Stroke Branch, National Institute of Neurological Disorders and Stroke, Bethesda, USA; 5 National Institute of Neurological Disorders and Stroke, National Institutes of Health, Bethesda, USA

**Keywords:** Endothelial progenitor cells, Endothelial cells, Flow cytometry, Cryopreservation, Angiogenesis

## Abstract

**Background:**

Endothelial progenitor cells (EPC) are markers of endothelial injury and may serve as a surrogate marker for vascular repair in interventional clinical trials. Objectives of this study were to modify a method of isolation of peripheral blood mononuclear cells (PBMC) and enumeration of EPC and mature endothelial cells (EC) from peripheral blood and to evaluate influence of cryopreservation on viability of PBMC and on numbers of EPC and EC.

**Patients/Methods:**

EPC and EC were analyzed in healthy volunteers in freshly isolated PBMC collected in CPT (cell preparation tubes) and in PBMC cryopreserved with: 1) Gibco Recovery^™^ Cell Culture Freezing Medium, 2) custom freezing medium. Viability of PBMC was tested using DAPI. EPC were gated for CD45^−^ CD34^+^CD133^+/−^VEGFR2^+/−^ and EC were gated for CD45^−^CD146^+^CD34^+/−^VEGFR2^+/−^.

**Results:**

Cryopreservation for 7 days at −80°C decreased viable PBMC from 94 ± 0.5% (fresh) to 84 ± 4% (the custom medium) and to 69 ± 8% (Gibco medium), while cryopreservation at −65°C decreased viability to 60 ± 6% (p<0.001, the custom medium) and 49 ± 5% (p<0.001, Gibco medium). In fresh samples early EPC (CD45^−^ CD34^+^CD133^+^VEGFR2^+^) were enumerated as 0.2 ± 0.06%, late EPC(CD45^−^CD146^+^CD34^+^VEGFR^2+^) as 0.6 ± 0.1% and mature EC (CD45^−^CD146^+^CD34^−^VEGFR2^+^) as 0.8 ± 0.3%of live PBMC. Cryopreservation with Gibco and the custom freezing medium at −80°C for 7 days decreased numbers EPC and EC, however, this decrease was not statistically significant.

**Conclusions:**

Our data indicate that cryopreservation at −80°C for 7 days decreases, although not significantly, viability of PBMC and numbers of subsets of EC and EPC. This method may provide an optimized approach to isolation and short-term cryopreservation of subsets of EPC and of mature EC suitable for multicenter trials.

## Background

Emerging evidence indicates that endothelial progenitor cells (EPC) may improve function of injured and ischemic organs by both induction and modulation of vasculogenesis [1,2]. EPC are markers of acute vascular damage and are elevated after ischemic stroke and traumatic brain injury (TBI), peaking at approximately day 7–14 after injury [3,4] and returning to baseline by 21–30 days [4,5]. In contrast, chronic vascular diseases (carotid atherosclerosis, diabetes, hypertension) [6,7], and cerebrovascular risk factors (smoking, depression, obesity and obstructive apnea) [8–10] are associated with decrease of numbers of EPC and their functional impairment.

Recent clinical trials show that transplantation of EPC may enhance myocardial angiogenesis [11], lead to functional improvement in peripheral vascular injury [2], and produce beneficial effects after brain ischemia (ClinicalTrials.gov identifier NCT00535197, NCT01468064). Traumatic brain injury (TBI) is the leading cause of death and disability in people under age 45 in industrialized countries [12,13]. Since circulating EPC can be augmented through a variety of pharmacologic interventions, they represent an attractive therapeutic target that may contribute to diminishing the size of TBI and to functional improvement, and EPC may serve as a surrogate marker of outcomes [14–16].

EPC originate from bone marrow and are recruited to sites of vessel injury while undergoing maturation to endothelial cells [17]. EPC represent only a tiny cell population in human peripheral blood [18]. Circulating EPC can be quantified in peripheral blood mononuclear cells (PBMC) by fluorescence-activated cell sorting (FACS) using specific cell-surface markers [19,20]. Despite extensive research, there is still a debate about the best definition of these cells [21,22]. However, EPC are generally identified by co-expression of various stem cell markers (CD34 and CD133) and endothelial cell markers (VEGFR2(vascular endothelial growth factor receptor marker)/(KDR or flk-1) [23], CD31(PECAM-1(Platelet endothelial cell adhesion molecule), CD144(VE(vascular endothelial)-cadherin [24]) in CD45-negative (lymphocyte common antigen) and CD14-negative (monocytes and macrophages) cell population [22,24,25]. While some protocols consider combinations of CD133^+^ (early progenitor marker), CD34^+^ (progenitor marker) and the VEGFR2/KDR^+^(endothelial marker) [26], other protocols use CD31^+^ (PECAM-1, endothelial cell marker) [18], or exclude early progenitor CD133 [21,27]. Numerous FACS protocols for enumeration of EPC are described [20,28] with various agreements between them [29,30]. However, currently “early EPC” with high proliferative capacity are defined by co-expression of two progenitor markers and of one endothelial marker (CD34^+^VEGFR2/KDR^+^CD133^+^) and considered to be a restrictive EPC phenotype [25]. At the same time “late EPC” with some proliferative potential can be characterized by co-expression of at least one progenitor marker and two endothelial markers(CD146^+^CD34^+^VEGFR2^+^) [31]. Circulating endothelial cells (EC) are mature differentiated cells which are shed from the vessel wall as a result of pathophysiological condition [32]. The consensus is that circulating EC are injured, dysfunctional or senescent cells [33] with low proliferative potential [34]. Circulating EC can be identified by expression of endothelial markers (CD146^+^ and VEGFR2/KDR^+^) without expression of stem cell markers (CD34^−^) [34]. Therefore, the first objective of this study was to develop a FACS protocol for enumeration of both “early” and “late” circulating EPC (as markers of progenitor potential) and EC populations (as injury severity marker) in human peripheral blood.

When clinical trials are performed at multiple centers, transport and storage of clinical specimens become important variables that may affect cell viability and modify numbers of subsets of EPC [35]. Cell isolation methods, freezing media, cell concentrations, freezing speed and storage conditions represent important variables in PBMC viability [35–37]. Current freezing protocols utilize various combinations of dimetyl sulfoxide (DMSO) and commercially available media, such as RPMI (Roswell Park Memorial Institute Medium), fetal bovine serum (FBS) and others [37-39]. There is a need to optimize the methods of cryopreservation of PBMC with evaluation of sample integrity and cell viability together with comparative analysis of subsets of EPC before and after cryopreservation. Therefore, the second objective was to evaluate influence of different freezing media on viability of PBMC and distribution of subsets of mature EC and EPC.

This report demonstrates that short-term cryopreservation decreases numbers of viable PBMC with use of both cryopreservation media. Cryopreservation for 7 days at −80°C decreases numbers of early and late EPC and of EC subsets determined in 3×10^6^ of live PBMC. However, compared to the fresh samples, the decrease of numbers of EPC subsets after cryopreservation was not statistically significant. Therefore, this method of isolation and cryopreservation of EPC and EC from human blood samples can be applied for clinical trials in the future.

## Materials and Methods

### Subjects and inform consent

Peripheral blood was obtained from healthy volunteers (N=16, age 52 ± 14 yrs (mean ± SD), 70% male) who donated blood in the Department of Transfusion Medicine (DTM), National Institutes of Health (NIH) under the protocol 99-CC-168 “Collection and Distribution of Blood Components from Healthy Donors for In Vitro Research Use”. The written informed consent was obtained from all participants before entering the study. Inclusion criteria were: 1) Age greater than or equal to 18 years and 2) weight greater than 110 pounds. Exclusion criteria were:1) No known heart, lung, kidney disease, or bleeding disorders; 2) No history of hepatitis since age 11; 3) No history of intravenous injection drug use in the past 5 years; 4) No receipt of clotting factor concentrates in the past 5 years; 5) No receipt of money or drugs in exchange for sex in the past 5 years; 6) No history of engaging in high-risk activities for exposure to the AIDS virus; and 7) Female subjects should not be pregnant. All donors were evaluated in pre-donation assessment visit, which included health history questionnaire, examination of blood pressure and heart rate, evaluation of arm veins, and blood tests to determine blood cell counts and detect exposure to HIV and hepatitis virus.

### Collection and processing of blood samples

Blood was collected in sodium heparin 8 ml CPT (cell preparation tubes, 16×125 mm, BD cat #362753), which were inverted 6–8 times and were centrifuged at 1800 g for 20 min at room temperature. Isolated peripheral blood mononuclear cells (PBMC) were counted using 0.4% of Trypan blue by cell counter (Cellometer^®^ auto T4 cell counter, Nexcelom) and aliquoted to contain 8×10^6^ live cells.

PBMC were analyzed either fresh (N=16), or were cryopreserved using one of the following conditions: 1) Gibco recovery^™^ cell culture freezing medium or 2) the custom freezing medium (62.5% fetal bovine serum (FBS) 10% Dimethyl sulfoxide (DMSO) in Lonza RPMI-1640 (12–702F)) (Table 1), for 7 days at temperature −65°C (N=8) and −80°C (N=8).

**Figure 1:**
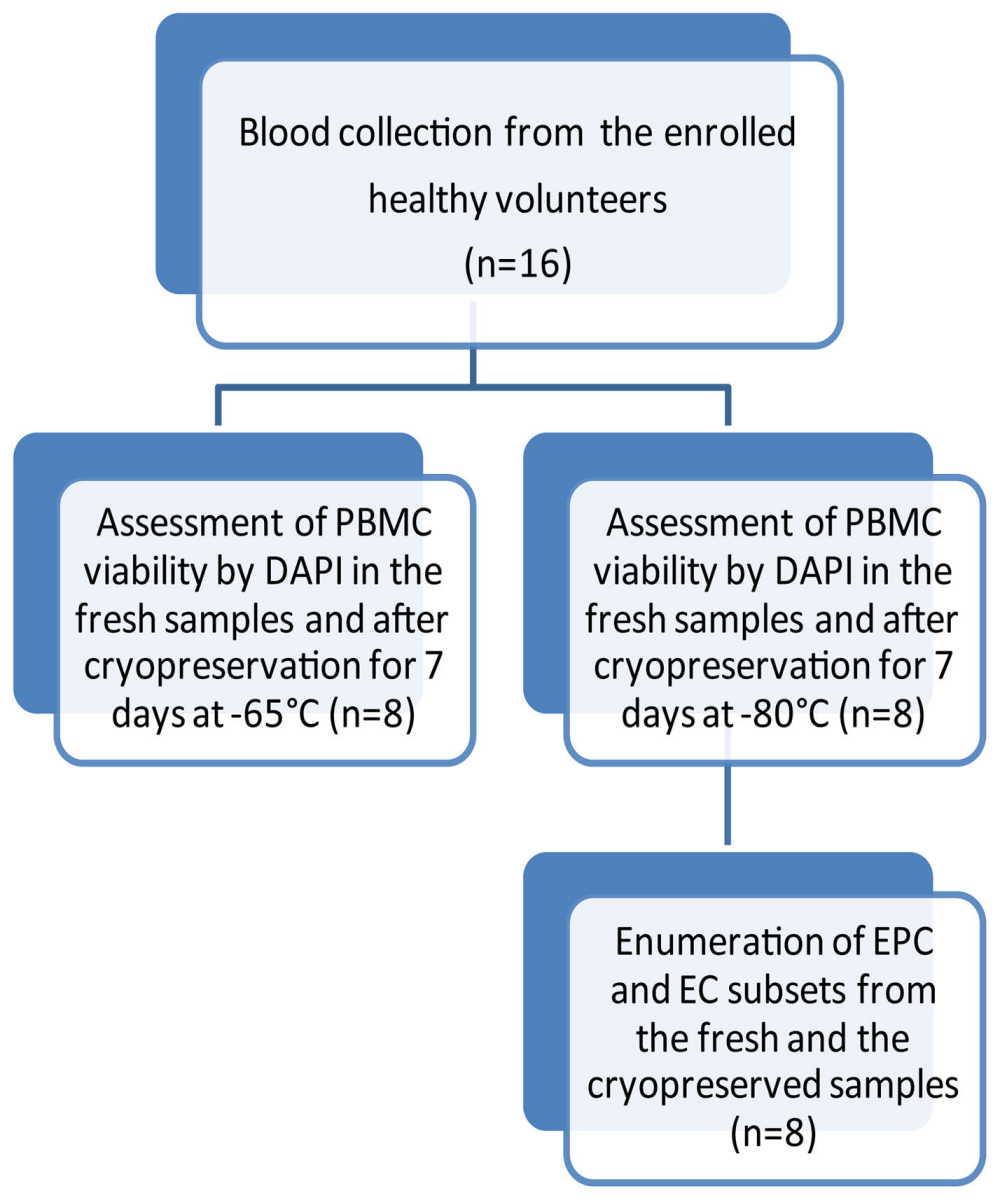
Overview of the sample collection and the procedures.

**Figure 2:**
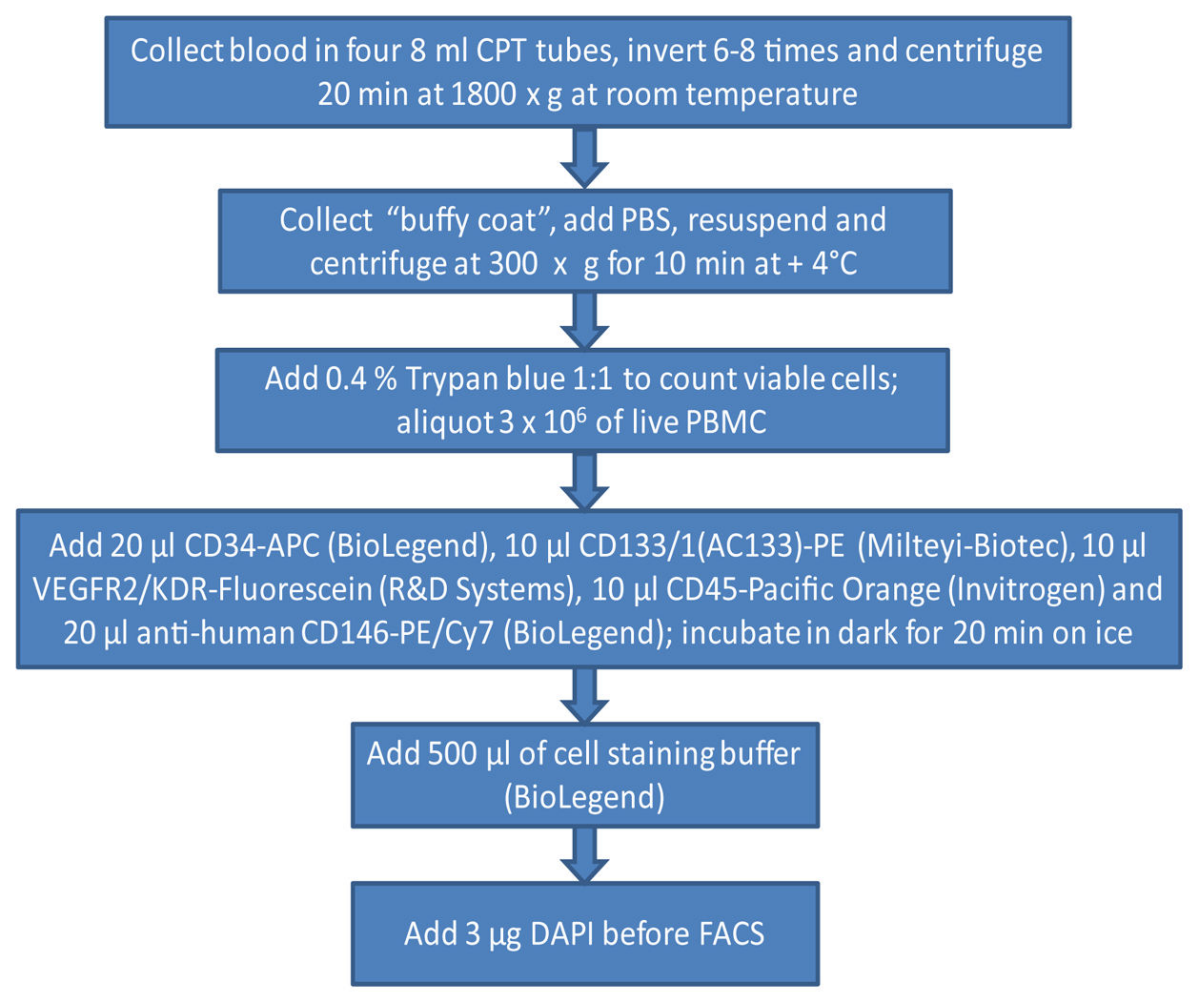
Flow chart of sample preparations.

For cryopreservation of isolated PBMC, the cells were resuspended in each freezing medium (Gibco or the custom medium) and then transferred to a pre-chilled 2 ml cryovials and were cryopreserved in a Nalgene freezing container providing a controlled freezing rate of 1°C/min and then were stored either −65°Cor at −80°C for 7 days prior to the FACS analysis (Figures 1 and 2).

Before staining, cryopreserved PBMC were quickly thawed in the add 37°C water bath (<2 min), washed in PBS, then the cells were counted with 0.4% of Trypan blue, and the staining procedures were performed, as described below.

### Staining protocol for EPC-EC measurements

For staining of fresh and cryopreserved EPC and EC, 3×10^6^ of live PBMC were incubated with 20 μl CD34-APC (BioLegend), 10 μl CD133/1(AC133)-PE (Milteyi-Biotec), 10 μl VEGFR2/KDR-Fluorescein (R&D Systems), 10 μl CD45-Pacific Orange (Invitrogen) and 20 μl anti-human CD146-PE/Cy7 (BioLegend) (all anti-human) with subsequent addition 500 μl of a cell staining buffer (BioLegend) and DAPI for assaying cell “viability”. Only fresh PBMC (N=16) or samples cryopreserved for 7 days at −80°C were used for EPC-EC measurements (Figure 2).

### Gating strategy

Cell enumeration by FACS (MoFloAstrios, Beckman Coulter) was performed immediately after the staining; all samples were kept protected from light. Just prior to the analysis, 1 μg of 4′,6-diamidino-2-phenylindole nuclear dye (DAPI, Invitrogen) per 1 million cells was added to the cell suspension to allow viability gating. To eliminate clumping, doublet discrimination was utilized as a part of the FACS procedure. From each sample, a limit of 1.000,000 events was set up. Events were gated for live cells, dead cells and subcellular debris (Sub). Live cells were gated for CD45-Pacific Orange negative cells and then further gated for CD34-APC positive cells. This subset was further gated for CD133^+^VEGFR2^−^, CD133^+^VEGFR2^+^, CD133^−^VEGFR2^−^, CD133^−^VEGFR2^+^ for enumeration of different EPC subsets. In addition to the EPC gating strategy, the live cells were negatively gated for CD45− Pacific Orange and were further gated for CD146^+^, which is used as a marker of mature EC. Subsequently, the CD146^+^ cells were gated on CD34^+^VEGFR2^−^, CD34^+^VEGFR2^+^, CD34^−^VEGFR2^−^ and CD34^−^ VEGFR2^+^ and designated as different EC subsets. For calculation of numbers of subsets of EPC the absolute numbers of each subset were divided by number of live PBMC and multiplied by 100.

For gating of EPC and EC the following isotype positive controls were used: APC Mouse IgG1, kappa-isotype control (FC) (BioLegend); mouse IgG1-PE (MACS MiltenyiBiotec); mouse IgG1, isotype control-CSF (R&D system); mouse IgG1-Pacific Orange (Invitrogen), PE/Cy7 Mouse IgG2a, kappa Isotype Ctrl (BioLegend).

### Data analysis

All instrument settings and the population gating methods were stored in a protocol file and remained unchanged throughout the analyses. Data analysis was performed using Kaluza flow cytometry analytical software (Beckman Coulter Inc., Brea, CA). Cell populations of EPCs and ECs were quantified as a percentage of live PBMC in each sample.

### Statistics

The Kolmogorov-Smirnov test was used for testing normality. Either parametric or non-parametric method was used depending on the data distribution for univariate data analysis. In addition, to examine a main effect of either temperature (fresh vs. cryopreservation at −80°C) or type of the medium (Gibco vs. the custom freezing medium) respectively, multivariate analysis of variance (MANOVA) models were used. In the MANOVA models, a compound symmetric covariance structure was employed in order to reflect possible correlations of the multiple measurements within a given subject. Only those EPC-EC subsets, which met the convergence criteria for fitting a MANOVA model, were reported. SAS version 9.2 or SPSS version 20.0 was used for data analysis. All statistical tests were two-sided and used a significance level of 0.05.

## Results

### Enumeration of EPC and EC subsets from human blood

**Figure 3:**
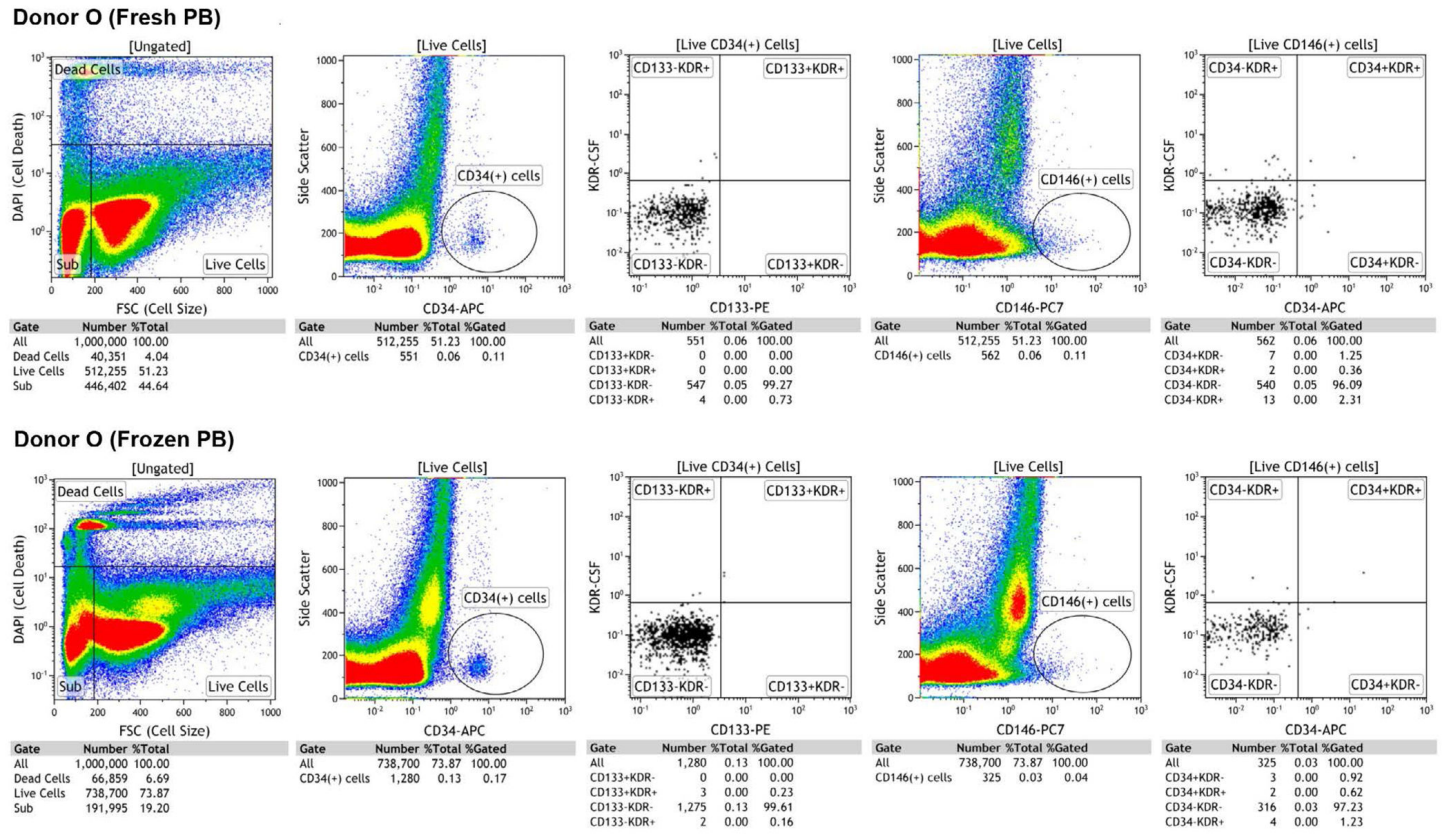
Enumeration of circulating EPC-EC. Example of gating strategy of EPC-EC by FACS. A representative FACS demonstrates enumeration of EPC and EC analyzed fresh and after cryopreservation. A. PBMC was assessed as viable by negative staining for DAPI. B. CD45+ cells were excluded and the rest of the cell population was further gated for CD34^+^ cells. C. CD34^+^ cells were gated for CD133 and VEGFR2 for enumeration of double and triple positive subsets of EPC. D. CD 45^+^ cells were excluded and live cells were further gated for CD146+. E. CD 146^+^ cells were further gated for CD34 and VEGFR2/(KDR) for enumeration of double and triple positive subsets of EC.

The gating strategy for EPC-EC is shown on Figure 3. Early and late EPC and EC subsets were enumerated from blood samples immediately after venipuncture followed by isolation, staining and subsequent FACS analysis of PBMC (n=8). Similar procedure was repeated after cryopreservation of PBMC for 7 days at −80°C (n=8), with thawing and staining using the same FACS protocol applied to fresh samples.

### Influence of cryopreservation at −65°C and −80°C for 7 days on PBMC viability

**Figure 4:**
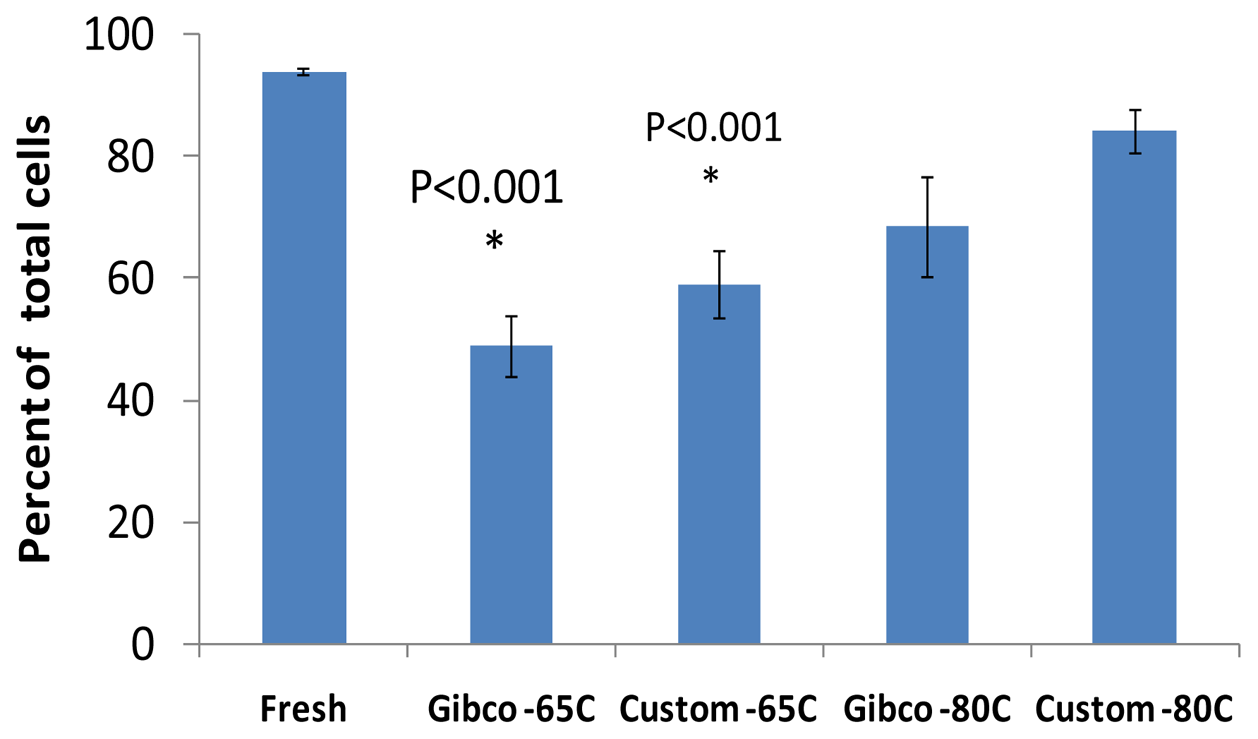
Influence of cryopreservation with Gibco Recovery^™^ Cell Culture Freezing Medium or the custom freezing medium at −65°C and −80°Cfor 7 days on PBMC viability assessed by FACS after addition of DAPI. Data is presented as mean ± SEM; *p<0.001 compared to fresh samples.

Samples of 16 participants were used for the PBMC viability study. Number of viable PBMC isolated using CPT tube was 0.83 ± 0.1×10^6^/ml of blood. Percentage of viable PBMC in the fresh human blood samples assessed by FACS using DAPI was 93.8 ± 0.5%. Cryopreservation of PBMC for 7 days at −80°C decreased viable PBMC from 94 ± 0.5% (fresh) to 84 ± 4% (the custom medium) and to 69 ± 8% (Gibco medium), albeit non-significantly. Though, cryopreservation PBMC for 7 days at −65°C further decreased viability 60 ± 6% (p<0.001, the custom medium) and 49 ± 5% (p<0.001, Gibco medium) compared to the fresh samples (non- parametric distribution of the data, Kruskall - Wallis test for both temperatures), (Figure 4).

### Influence of cryopreservation for 7 days at −80°C on numbers of double positive and triple positive subsets of EPC and EC

Early EPC (CD34^+^CD133^+^VEGFR2^+^) were enumerated as 0.2 ± 0.06%, late EPC (CD146^+^CD34^+^VEGFR2^+^) as 0.6 ± 0.1% and mature EC (CD146^+^CD34^−^VEGFR2^+^) as 0.8 ± 0.3% of live PBMC in fresh samples. Because cryopreservation at −65°C for 7 days resulted in significant decrease of numbers of viable PBMC (Figure 4), the EPC analysis was carried only on samples cryopreserved at −80°C for 7 days (N=8). The numbers of early and late EPC and EC cryopreserved with Gibco medium and with the custom freezing medium at −80°C are presented in Table 2, (n=8).

Short-term cryopreservation for 7 days with both Gibco and the custom medium decreased numbers of early and late EPC and EC. Cryopreservation with the Gibco medium resulted in slightly higher numbers of EPC- EC compared to those cryopreserved with the custom medium. However, no significant difference (Kruskall-Wallis test) was found in the numbers of EPC-EC subsets from samples cryopreserved with the Gibco and with the custom medium compared to those enumerated from the fresh samples. Likewise, MANOVA did now show the effects of temperature, cryopreservation at −80°C, and type of the medium (Gibco or the custom medium) on the numbers of EPC-EC subsets (Table 3). Although cryopreservation for 7 days at −80°C decreased numbers of EPC and EC, the decrease was not statistically significant.

## Discussion

This study shows that short-term cryopreservation of human PBMC at −80°C decrease numbers of subsets of EC and EPC expressed as percentage of viable PBMC. Successful preservation of EPC is a main prerequisite for evaluation of their potential as surrogate marker of vascular injury and repair, particularly, in interventional clinical trials. However, to date no efficient cryopreservation protocols for EPCs are available which would fulfill the requirements for multicenter trials. These protocols should include collection of samples in various hospitals, prompt isolation of cells, sera or plasma with subsequent cryopreservation for short-term storage and further transportation to a central biorepository for long-term storage and appropriate analysis. Here we present the results on short-term cryopreservation of PBMC and on effects of two freezing media on viability of EPC and EC derived from human peripheral blood.

The major purpose was to establish optimal protocols for both enumeration and short-term cryopreservation of EC and EPC, which may be appropriate for clinical trials on stimulation of angiogenesis which will be conducted in the future. EPC are a subtype of stem cell with high proliferative potential capable of differentiating into mature endothelial cells and contributing to neovascularization [21,22,28]. The definition, identification and characterization of EPC are still evolving [21]. Circulating EC are differentiated cells shred from the endothelium [17,19,32]. Increased EC levels are determined in endovascular procedures, sickle cell anemia [40], infections, myocardial infarction [41], cancer [42] and may serve as injury marker [32]. Hence the purpose of this work was to develop a method which is able to simultaneously quantify both non-mature progenitors (as markers of regeneration) and endothelial cells (as markers of injury). In addition, we examined influence of cryopreservation of PBMC and of various freezing media on EPC numbers.

The technique of isolation of PBMC utilizes CPT [32], which is advantageous for blood processing for clinical trials due to minimizing steps in isolation of EPC. In our study, use of CPT tubes resulted in viability of PBMC in fresh samples up to 94%. This is comparable to findings of Ruitenberg showing range of viability 85–99% of PBMC in fresh samples after CPT preparation [32].

Our data show better cell viability of PBMC after cryopreservation with the medium containing high 62.5% FBS and 10% DMSO in RPMI (the custom medium) compared to high glucose and low FBS concentration (Gibco cryopreservation medium). Plasma proteins have cryoprotectant effects, most probably by modifying the viscosity of the cryoprotective solution [36]. Because of high content of embryonic growth promoting factors, FBS has been successfully used for cryopreservation of progenitor cells [37]. Our findings are in line with previous results showing optimal viability of porcine progenitor cells stored of 50% FBS with 10% DMSO up to 18 months at −165°C [37].

Both cryopreservation media used in the study contained 10% (v/v) of DMSO, which is optimal and consistent with the most cryopreservation protocols. Current cryopreservation methods utilize various cytoprotectants and to retain their function after cryopreservation [36,37,43]. DMSO is the most widely used cryoprotective agent for human PBMC, which prevents intracellular ice crystal formation [36]. Even low (5–7.5% (v/v)) DMSO concentrations show good recovery of human CD34^+^ cells [36,44]. However, addition of DMSO at higher concentrations (20%) may increase cell clumping [45] and further increase in concentration (40%) may have deleterious osmotic and toxic effects on cryopreserved cells [46].

The results of our study demonstrate that cryopreservation for 7 days decreased, albeit non - significantly numbers live PBMC after cryopreservation at −80°C. In actual local hospital settings enrolling patients to clinical trials, the temperature of freezers may vary with deviation of 10°C from the optimal. Our data show that short-term cryopreservation at −65°C further decreased viability of PBMC compared to the viability determined after storage at −80°C and the difference with fresh samples became significant. This further support critical importance of preserving temperature of even short-term cryopreservation optimized to at least to −80°C for further accurate EPC measurements.

The limitations of the study are the relatively high temperatures (−65°C, −80°C) and short time (7 days) used for cryopreservation of PBMC. Frozen cell lines are stable at −80°C for a few weeks [36]. On the other hand, storage in liquid nitrogen at −160°C blocks enzymatic pathways in the cell, permitting long-term storage [36]. However, some studies showed that PBMC can be stored with 5% DMSO at −80°C with good recovery 16 months later [39], and hematopoietic cell can be preserved at −80°C in 5%–10% DMSO with 80% viability up to 12 months [43]. Local hospitals enrolling patients into clinical trials may not be always equipped with liquid nitrogen facilities allowing cryopreservation and storage of samples at −160°C, and this study was designed to evaluate conditions of cryopreservation appropriate for multicenter clinical trials. Cryopreservation for 7 days at −80°C will be sufficient either to transfer samples to long-term liquid nitrogen storage −160°C, or to arrange enumeration of EPC-EC counts by FACS in current laboratory settings. We found that the custom medium containing high concentration of FBS resulted in better PBMC viability than Gibco medium (84 ± 4% vs. 69 ± 8%), although this difference was not statistically significant. However, formally we did not find significant influence of type of freezing media on EPC-EC numbers during short-term cryopreservation, a consistent type of freezing media should be used for all samples collected during the study. Further studies are warranted to establish recovery rate and viability of human EPC-EC after long-term cryopreservation.

The challenge of EPC analysis is related to low numbers of subsets of EPC in peripheral blood and to high variability of the subsets among the normal population. Our previous data show that at least 3.000,000 events FACS events can be collected from 32 ml of blood [15]. For comparative purpose we chose the fixed experimental design with number 3×10^6^ of live PBMC in the sample and with FACS acquisition with limit of 1.000,000 of events. This resulted in relatively low numbers of EPC and especially in decrease of rare subset of early EPC CD34^+^CD133^+^VEGFR2^+^ in preparation with the custom medium. Other studies showed low numbers of this subset in peripheral blood [25]. However, decrease of CD34^+^CD133^+^VEGFR2^+^ was observed after cryopreservation with Gibco medium as well, and can be explained by susceptibility of this subset to cryopreservation. In our study the numbers of EPC subsets were expressed as percentage of absolute numbers in live PBMC, and this way was also chosen to eliminate influence of covariates. Recently we enumerated subsets of EPC from human blood using fixed numbers of live PBMC ranged from 1×10^6^ to 10×10^6^ expressed per ml of blood (Bogoslovsky, unpublished data). We found good correlation between the numbers of EPC subsets expressed per ml of blood, with decrease of variability proportional to the increase of numbers of PBMC used for the experiments.

In conclusion, this report indicates that cryopreservation at −80°C for 7 days decreases, although not significantly, viability of PBMC and numbers of subsets of EC and EPC. The field of enumeration and characterization of EPC as outcome measures for clinical trials is constantly evolving, and further studies are warranted.

## Conclusions

The method of short-term cryopreservation with sufficient viability makes this protocol valuable for multicenter clinical trials aimed to increase EPC levels and to promote neovascularization. Our optimized flow cytometry protocol enables quantitative rapid enumeration of subsets of EPC and EC. We believe that this optimized version of the EPC assay should have clinical relevance, since accumulating evidence advises the monitoring of circulating EPC and EC as novel surrogate prognostic and diagnostic cellular biomarkers.

## Figures and Tables

**Table 1:** Formulations of the Gibco recovery medium and the custom medium used for cryopreservation of PBMC for 7 days at −65°C and −80°C.

Gibco Medium	Custom Medium
High-glucose Dulbecco’s Modified Eagle Medium (DMEM) −80% Contains high glucose, non-essential amino acids, sodium pyruvate, phenol red Bovine Serum and FBS −10% DMSO-10%	RPMI-1640 (12–702F)-27.5% Contains sodium bicarbonate (NaHCO_3_) 26.7 ml/L of 7.5% NaHCO3 and 10.3 ml of 200 mM L-glutamine FBS-62.5% DMSO-10%

**Table 2:** Subsets of EPC and EC from viable PBMC isolated from human blood (n=8) (Mean ± SEM). Data are expressed as percent of subsets per number live PBMC from the fresh samples and after cryopreservation for 7 days at −80°C (Gibco and the custom freezing medium). NS using Kruskall-Wallis test.

PBMC phenotype	Proposed Function	Fresh Sample	Cryopreserved with Gibco medium	Cryopreserved with the custom medium	Kruskall-Wallis Statistics
CD34+CD133+VEGFR2−	“Early EPC”	0.3 ± 0.07	0.3 ± 0.1	0.3 ± 0.2	P<0.96
CD34+CD133+VEGFR2+	“Early EPC”	0.2 ± 0.06	0.09 ± 0.05	0.00 ± 0.00	P<0.17
CD34+CD133−VEGFR2+	“Early EPC”	0.6 ± 0.1	0.5 ± 0.2	0.2 ± 0.02	P<0.21
CD146+CD34+VEGFR2−	“Late EPC”	1.1 ± 0.2	1.4 ± 0.4	1.6 ± 0.6	P<0.67
CD146+CD34+VEGFR2+	“Late EPC”	0.6 ± 0.1	0.4 ± 0.2	0.3 ± 0.1	P<0.26
CD146+CD34−VEGFR2+	“EC”	0.8 ± 0.3	0.6 ± 0.1	0.2 ± 0.1	P<0.07

**Table 3:** MANOVA showing no significant influence of cryopreservation for 7 days −80°C and type of medium (Gibco or the custom medium) on cell subsets EPC-EC (double or triple positive subsets expressed as percentage in live PBMC).

PBMC phenotype	Type of medium	Temperature

CD34+CD133+VEGFR2−	F_2, 9_=0.42, p=0.6714	F_1, 7_=0.07, p=0.7946
CD34+CD133−VEGFR2+	F_2, 9_=0.49, p=0.6280	F_1, 7_=0.87, p=0.3831
CD146+CD34+VEGFR2−	F_2, 9_=0.91, p=0.4367	F_1, 7_=1.40, p=0.2746
CD146+CD34+VEGFR2+	F_2, 9_=0.49, p=0.6280	F_1, 7_=1.05, p=0.3394
CD146+CD34−VEGFR2+	F_2, 9_=1.61, p=0.2521	F_1, 7_=1.55, p=0.2533

## Abbreviations

DMSO: Dimethyl Sulphoxide
EPC: Endothelial Progenitor Cells
EC: Circulating Endothelial Cells
FBS: Fetal Bovine Serum
PBMC: Peripheral Blood Mononuclear Cells
DTM: Department of Transfusion Medicine
TBI: Traumatic Brain Injury
CPT: Cell Preparation Tube
PECAM-1: Platelet Endothelial Cell Adhesion Molecule
VE-cadherin: Vascular Endothelial-Cadherin
